# Removal of Ibuprofen at Low Concentration Using a Newly Formulated Emulsion Liquid Membrane

**DOI:** 10.3390/membranes11100740

**Published:** 2021-09-28

**Authors:** Abdul Latif Ahmad, Mohd Hazarel Zairy Mohd Harun, Mohd Khairul Akmal Jasni, Nur Dina Zaulkiflee

**Affiliations:** School of Chemical Engineering, Engineering Campus, Universiti Sains Malaysia, Nibong Tebal 14300, Pulau Pinang, Malaysia; mmohazarel@gmail.com (M.H.Z.M.H.); jasnikhairul22@gmail.com (M.K.A.J.); nurdinazaulkiflee@gmail.com (N.D.Z.)

**Keywords:** ibuprofen (IBP), emulsion liquid membrane (ELM), low concentration

## Abstract

Ibuprofen (IBP) is a pharmaceutical product that is widely prescribed as an over-the-counter painkiller. It has been classified as a contaminant of emerging concern (CEC) that has received global attention in the search for a better wastewater separation technology. The emulsion liquid membrane (ELM) is one of the potential solutions for IBP removal from wastewater owing to its advantages, such as the ability to remove a highly soluble solute, energy efficient and tuneable formulation. To develop this ELM, a series of parameters such as stirring speed, emulsification time, organic to internal phase volume ratio (O/I), internal phase concentration, carrier concentration and surfactant concentration were studied. The extraction was carried out for 15 min stirring time and the concentration of IBP in the feed phase was determined using a UV-Vis spectrophotometer. The optimum formulation for the ELM was found at 300 rpm stirring speed, 20 min emulsification time, 3:1 of O/I, 0.1 M ammonia, NH_3_ (stripping agent), 6 wt% trioctylamine, TOA (carrier) and 2 wt% sorbitan monooleate, Span 80 (non-ionic surfactant). IBP removal of 89% was achieved at the optimum parameters of ELM. The current research demonstrated that a newly formulated ELM has great potential in removing a low concentration IBP from wastewater.

## 1. Introduction

Ibuprofen (IBP) is a non-steroidal anti-inflammatory drug (NSAID) that works by inhibiting the action of cyclooxygenase (COX) enzyme to synthesize prostaglandin, which causes inflammation in living organisms. With the fleet rise of 21st century inflammatory diseases such as fever, osteoarthritis and headaches, the flourishing growth of the IBP market is inescapable by 2027 [1]. This drug is also essential for the preservation of livestock and aquatic organisms in the agriculture and aquaculture sectors, respectively [2,3].

Despite its benefits, IBP is classified as a contaminant of emerging concern (CEC) that can harm the environment and living organisms in view of its toxic pharmaceutically active compounds found in water bodies [4]. The absence of IBP discharge threshold imposed on industrial and health sectors has allowed them to simply excrete the partially treated wastewater, thus causing the accumulation of IBP in the stream [5]. Chopra et al. reviewed that the presence of this drug and its residual in water systems, even at low concentrations, threatens the quality and safety of drinking water [6]. This is because, apart from antibiotics, the presence of IBP in most water treatment plants (WTPs) in many countries is also the source for antimicrobial resistance in the population, which has been yet overlooked [7,8,9]. Horribly, consumption of this contaminated water by humans and animals will raise concerns regarding bacterial resistance, which increases the mortality rate since infections will be intractable. Not to mention, the presence of IBP in the aquatic ecosystem has also contaminated non-targeted fish, resulting in the growth inhibition and inactivation of functional enzymes in range of aquatic organisms [6,10].

Until now, the separation of IBP has presented a major challenge in wastewater treatment plants (WWTPs), owing to their poor degradability and low detection limit [6,11]. IBP’s water solubility of 58 ppm, which is relatively higher than diclofenac and naproxen, has allowed it to be easily transported from one water matrix to another, lowering adsorption during sewage treatment [12]. Consequently, the IBP residual remained in the sludge and, to some extent, caused high ecotoxicological impacts towards the terrestrial ecosystem following the presence of IBP during application of sewage sludge for agriculture. Advanced methods such as ozonation [13], advanced oxidation processes (AOPs) [14] and Fenton oxidation [15] appeared to be ineffective due to secondary pollution generation, high energy requirements, usage of high-cost reagents and system complexity. It has been reported that 50 ppm IBP was treated with a reactive oxidising agent and demonstrated a low degradation rate of up to 57.33% after 90 min of irradiation [14]. By utilising an ozone system and Fe^3+^, the removal efficiency of 0.1 mM IBP was approximately 50% [16]. As a result, these methods were unable to successfully treat low IBP concentrations, and their performance had deteriorated over time.

**Figure 1:**
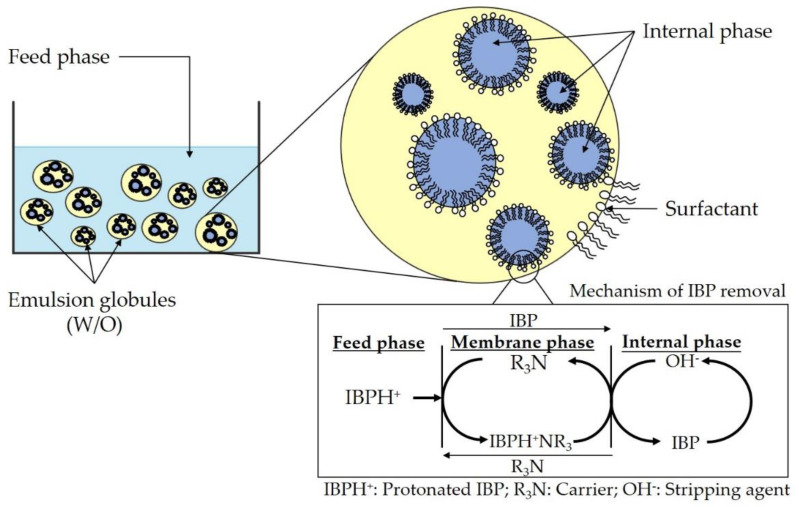
ELM mechanism for pollutant removal.

To overcome this limitation, a superior advanced separation process must be further investigated. Therefore, emulsion liquid membrane (ELM) is one of the potential technologies to remove a persistent pharmaceutical contaminant from wastewater. ELM is distinguished by its ability to simply extract a specific micropollutant using a tunable chemical formulation with minimal energy consumption [17,18,19]. Theoretically, there are three phases required to develop this ELM, known as a double emulsion, water-in-oil-in-water (W/O/W) system, such as stripping phase, membrane phase and feed phase. Figure 1 shows the mechanism of this double emulsion system which involves the extraction of pollutant from the feed phase by a carrier, formation of a carrier-pollutant complex in the membrane phase and pollutant stripping in the internal phase. ELM is a single-step process that applied a liquid–liquid extraction principle where the extraction and stripping occurred concurrently in order to concentrate and purify the desired solute [20].

Pharmaceutical pollutants such as ethyl-paraben (20 ppm) [21], diclofenac (60 ppm) [22], amoxicillin (71 ppm) [23] and tetracycline (100 ppm) [24] could be successfully extracted using different formulations of ELM. It was reported by Razo-Lazcano et al. that the extraction efficiency of 50 ppm IBP was around 100% at a stirring speed of 8000–13500 rpm [25]. However, despite the discovery, the use of a high feed phase concentration at high stirring speed had potentially decreased the ELM performance for a low solute concentration as well as inefficient energy consumption during treatment. Conversely, the common use of stripping agents such as HCl, Na_2_CO_3_, H_2_SO_4_, HNO_3_ and NH_3_ in the internal phase may have contributed to the pollutant removal efficiency. By using HCl, Na_2_CO_3_ and H_2_SO_4_ in the formulation, for example, the percentage of phenol removed was less than 40% [26]. The formulation of ELM using NH_3_ (internal phase), TOA (carrier) and kerosene (membrane phase) was reported to achieve 80% removal of acetaminophen (antibiotics) from feed phase [18].

Therefore, ELM is a promising technology to separate pharmaceutical compounds in view of the simple mechanism and formulation to extract low feed phase concentration. To the best of our knowledge, no experimental work has been conducted to extract low concentrations of IBP by using an ELM formulation that consists of kerosene and NH_3_ as its membrane and internal phase, respectively. Thus, the current research is attempting to elucidate the potential of a new ELM formulation to remove a low IBP concentration in wastewater. The optimum condition would be investigated such as the ELM formulation which includes the composition variation of the carrier, surfactant and stripping agent under optimal emulsification and stirring speed in obtaining a high removal efficiency of IBP. Ultraviolet-visible (UV-Vis) spectroscopy was used to evaluate the removal efficiency.

## 2. Experimental

### 2.1. Chemicals

Kerosene (Sigma Aldrich, St. Louis, MO, USA) and triocytlamine, TOA (Merck, Darmstadt, Germany) were prepared as organic diluent and carrier, respectively. For the membrane and internal phase, sorbitan monooleate, Span 80 (Merck, Hohenbrunn, Germany) was used as a non-ionic surfactant, and ammonia, NH_3_ (Merck, Darmstadt, Germany) was employed as a stripping agent (internal phase). Ibuprofen, IBP (Sigma Aldrich, St. Louis, MO, USA) was obtained as a pollutant model while hydrochloric acid, HCl (Merck, Darmstadt, Germany) was used to alter the pH in the feed phase. All the chemicals used were analytical grade. 

### 2.2. Emulsion Preparation

**Figure 2:**
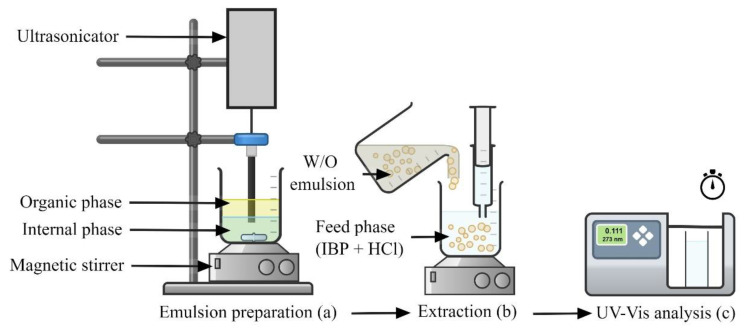
Schematic diagram of (**a**) emulsion preparation; (**b**) IBP extraction; (**c**) UV-Vis analysis.

The ELM double emulsion system was created through the emulsification process. For the first emulsion, water-in-oil emulsion (W/O), it was prepared by generating the membrane and internal phase. The membrane phase was made by mixing the kerosene (diluent) with a particular amount of TOA (carrier) and Span 80 (non-ionic surfactant). The membrane phase and NH_3_ (internal phase) were then stirred gently by using a magnetic stirrer (IKA, Staufen, Germany) with an Ultrasonicator (Telsonic Ultrasonix, Mumbai, India) to form a W/O emulsion. Table 1 displays the chosen experimental conditions for this project. Figure 2 shows the setup of the experiment begin with the emulsion preparation followed by extraction and UV-Vis analysis. 

### 2.3. Analytical Extraction

**Figure 3:**
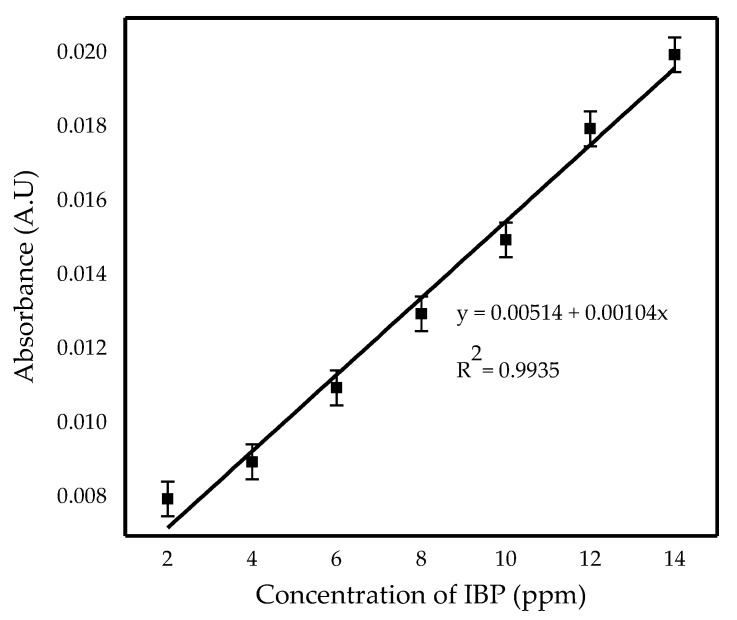
Calibration curve UV-Vis absorbance against concentration of IBP (ppm).

A feed phase contained IBP and 0.1 M of HCl was used as the pollutant model for the whole experiment. The prepared (W/O) emulsion described in Section 2.2 was then dispersed in the feed phase and stirred by using a magnetic stirrer. After a few minutes of stirring, the maximum absorbance of IBP in feed solution was measured using a UV-Vis spectrophotometer (Spectroquant Pharo 300, Merck, Darmstadt, Germany). Prior to the investigation, a relationship between the IBP concentration and absorbance needed to be constructed. Thus, the initial concentration of IBP in the feed phase was varied from 2–14 ppm. Figure 3 shows the calibration curve (linear-fit) of absorbance against IBP concentration.

From its linear equation in Figure 3, the final concentration, C_i_ of the feed phase was determined corresponding to its maximum absorbance. According to the literature, the wavelength corresponding to the maximum IBP absorbance is detected at 273 nm [27]. The removal efficiency was used to calculate the amount of IBP removed, as shown in the following Equation (1):

(1) $$\mathrm{Removal}\;\mathrm{efficiency}\;\left(\%\right)=\frac{C_{o}-C_{i}}{C_{o}}\times 100\%$$

where C_o_ is the initial concentration of IBP in the feed phase at 10 ppm while C_i_ is a final concentration of IBP in the feed phase (ppm).

### 2.4. Transport Mechanism of IBP

Both the extraction and stripping processes of IBP followed the Type II facilitated transport as elucidated in equilibrium Equations (2) and (3), respectively. It was reported by Dâas et al. that the addition of acidic and basic solution in the feed phase can manipulate the IBP towards protonated and deprotonated form [20]. Hence, the addition of HCl in the feed phase had protonated the IBP into IBPH^+^. During the extraction process, the IBPH^+^ formed an ion-complex pair with the carrier (TOA) at the feed-membrane interface, as shown in Equation (2). In this chemical equation, the TOA was simply noted as R_3_N. Meanwhile, in the stripping process, the ion-complex diffused at the membrane-internal interface and chemically stripped by ammonia, as shown in Equation (3).

Feed-membrane interface


(2) $$\left[{\mathrm{IBPH}}^{+}\right]\;+\;\left[R_{3}N\right]\;↔\;\left[{\mathrm{IBPH}}^{+}{\mathrm{NR}}_{3}\right]$$

Membrane-internal interface

(3) $$\left[{\mathrm{IBPH}}^{+}{\mathrm{NR}}_{3}\right]\;+\;\left[{\mathrm{OH}}^{-}\right]\;↔\;\left[\mathrm{IBP}\right]\;+\;\left[R_{3}N\right]\;+\;\left[H_{2}O\right]$$

## 3. Results and Discussion

### 3.1. Effect of Stirring Speed during Emulsification

**Figure 4:**
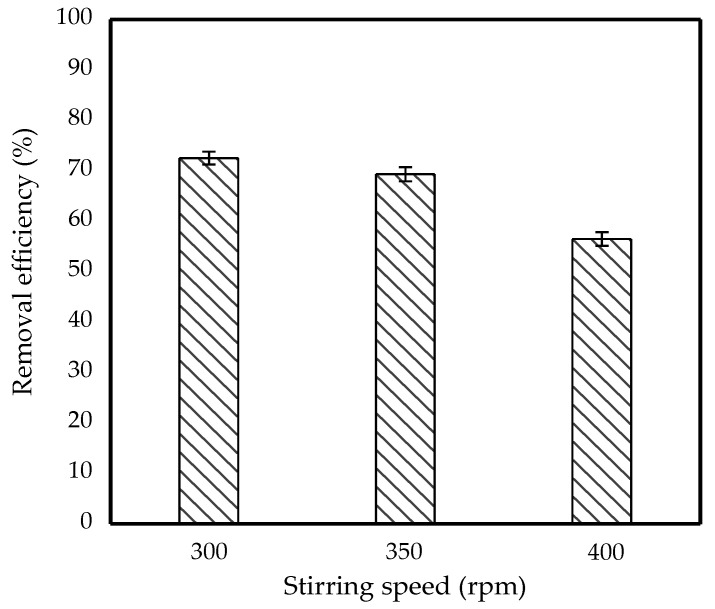
Effect of stirring speed on removal efficiency of 10 ppm IBP. (Conditions: [TOA] = 6 wt%; [Span 80] = 6 wt%; Emulsification time = 15 min; O/I = 3:1, Kerosene = 30 mL).

The effect of stirring speed on the separation of IBP from the feed phase was investigated by stirring the solution at 300–400 rpm with a 50 rpm increment. Figure 4 displays the removal efficiency of IBP at different stirring speeds. As depicted in Figure 4, the removal efficiency decreases as the stirring speed increases. At 300 rpm, for example, nearly 73% of the IBP was removed, whereas at 400 rpm, it was only 56%. This is attributed to the reason that stirring intensity is one of the factors that contributes to the emulsion’s shear stress and uniformity. At a low stirring speed, the emulsion dispersion was uniformed in the feed phase which indicated a low shear stress of the emulsion which results in a high mass transfer of IBP into the membrane phase.

However, as the stirring speed increased, the removal efficiency had progressively decreased. This phenomenon could be explained by Fouad et al., who reported that aggressive stirring will cause a high shear force applied on the emulsion which leads to rupture of the globules into small droplets [28]. According to Ahmad et al., the rupture affected the emulsion stability since the emulsion was unable to withstand the shear forces, resulting in the internal spillage and leading to a partial of pollutant in the feed phase which was recovered during the extraction process [29]. Thus, a gentle mixing at 300 rpm was required to achieve high IBP removal efficiency.

### 3.2. Effect of Emulsification Time

**Figure 5:**
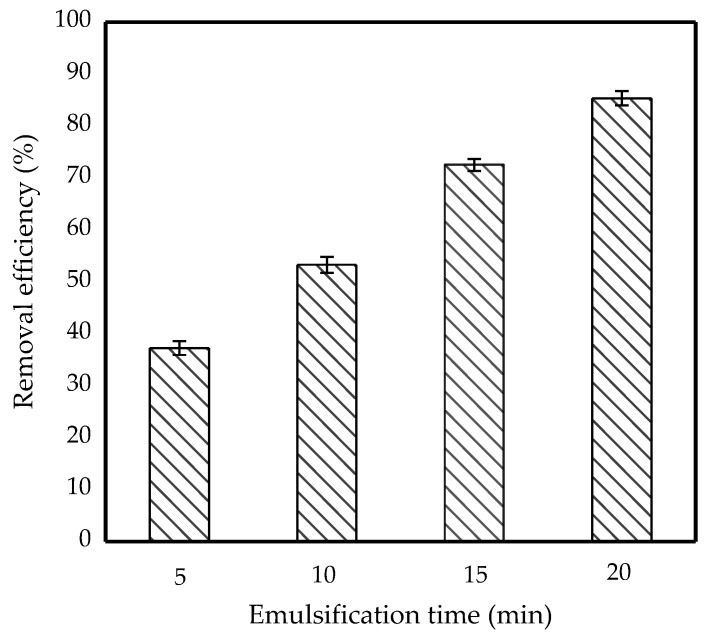
Effect of emulsification time on removal efficiency of 10 ppm IBP. (Condition: [TOA] = 6 wt%; [Span 80] = 6 wt%; Stirring speed = 300 rpm; O/I = 3:1; Kerosene = 30 mL).

The removal efficiency was strongly influenced by the emulsification time. Therefore, the emulsification process was varied for 5, 10, 15 and 20 min to investigate its effect on IBP removal. Figure 5 shows the bar chart profile of the removal efficiency at different emulsification times.

Initially, after 15 min of emulsification, the emulsion was ready and IBP removal was completed with a 73% efficiency. Increasing the emulsification time to 20 min interestingly increased the removal efficiency to 85%. However, emulsification time below 15 min reduced the removal efficiency. It was reported that the time required to emulsify will significantly impact the size of the globule [30]. A 20-min emulsification time resulted in more evenly distributed small globules, whereas a shorter time led to an unequal diameter of small globules. The small globules increased the mass transfer area for the IBP to diffuse through the membrane. Thus, a longer emulsification time was critical to achieve high IBP removal efficiency from the feed phase.

### 3.3. Effect of Organic to Internal Phase Volume Ratio (O/I)

**Figure 6:**
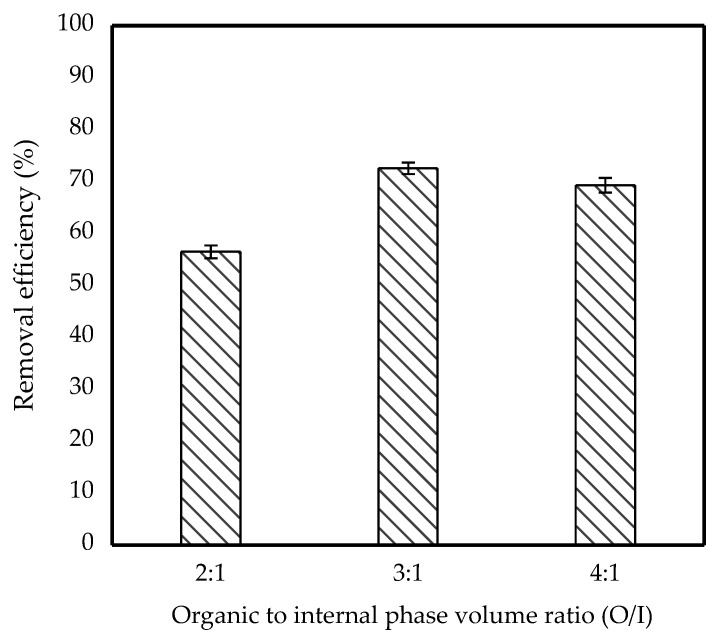
Effect of O/I ratio on removal efficiency of 10 ppm IBP. (Condition: [TOA] = 6 wt%; [Span 80] = 6 wt%; Stirring speed = 300 rpm; Emulsification time = 20 min; Kerosene = 30 mL).

The effect of O/I ratio on IBP removal efficiency was studied, and the results are shown in Figure 6. Organic volume of kerosene was changed while the volume of the internal phase remained constant, resulting in investigation ratios of 2:1, 3:1 and 4:1. It shows that the removal efficiency of IBP differed depending on the organic to internal phase volume ratio, which is in the order of 2:1 < 4:1 < 3:1.

The highest removal efficiency of 73% at a 3:1 ratio indicated that IBP was removed more effectively from the feed phase. This is because a larger volume of organic phase corresponded to a thicker emulsion wall, indicating the production of a highly stable emulsion. However, a further increase in organic volume beyond 3:1 had caused a slight decrease in the removal efficiency to 69% due to an increase in the barrier between feed and emulsion droplets which resulted in low pollutant mass transfer [20]. In addition, an excessive organic volume will cause insufficient surfactant to reduce surface tension between the aqueous and organic phases, resulting in poor emulsion droplet dispersion [30]. Meanwhile, below a 3:1 ratio, the removal efficiency was the lowest due to insufficient organic phase volume which resulted in a thin emulsion wall and caused the emulsion to break [31]. Thus, a 3:1 ratio was thought to be the most effective for removing high amounts of IBP from the feed phase.

### 3.4. Effect of Stripping Agent Concentration

**Figure 7:**
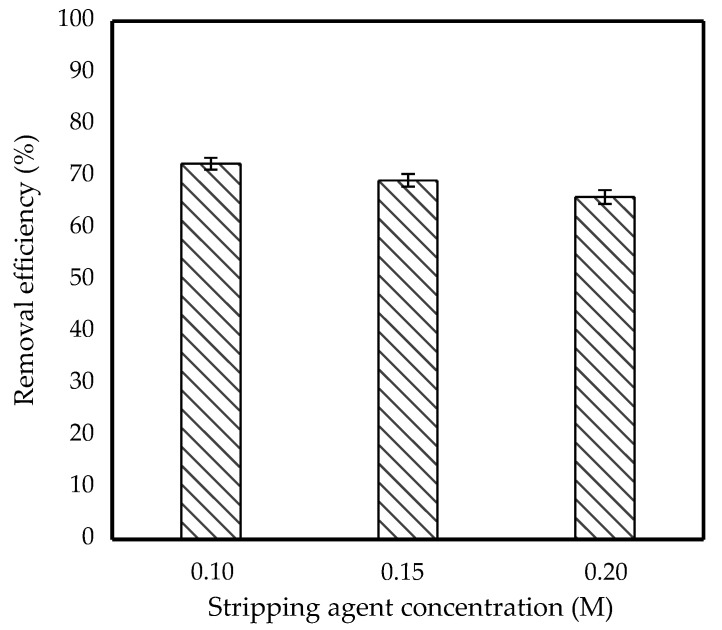
Effect of stripping agent concentration on removal efficiency of 10 ppm IBP. (Condition: [TOA] = 6 wt%; [Span 80] = 6 wt%; Emulsification time = 20 min; Stirring speed = 300 rpm; O/I = 3:1; Kerosene = 30 mL).

Figure 7 shows the effect of varying stripping agent concentrations on IBP removal efficiency. For this experiment, 0.1 M, 0.15 M and 0.20 M NH_3_ solution concentrations were chosen. The removal efficiency decreased as the concentration of NH_3_ solution increased, as shown in the graph. According to Kulkarni et al., emulsion swelling was strongly influenced by the basicity of the internal phase, which induced a significant osmotic pressure difference between the feed and the internal phase [32]. In other words, a high concentration of NH_3_ provided high moles of OH^−^ and resulted in rapid permeation of IBP into the stripping phase, causing globule size to increase. The tendency of the emulsion to break increased as the volume of globules increased, hence lowering the IBP removal efficiency. As a result, an optimal NH_3_ amount of 0.10 M was considered the optimum stripping condition to achieve high IBP removal.

### 3.5. Effect of Carrier Concentration

**Figure 8:**
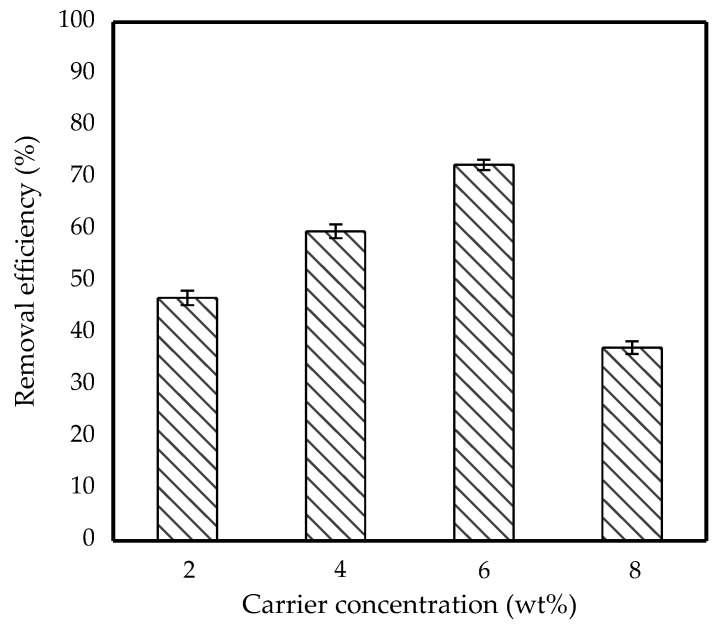
Effect of carrier concentration on removal efficiency of 10 ppm IBP. (Condition: [Span 80] = 6 wt%; Stirring speed = 300 rpm; Emulsification time = 20 min; O/I = 3:1; Kerosene = 30 mL).

The effect of different concentrations of carrier on removal efficiency was investigated and the result is shown in Figure 8. As the concentration of TOA increased from 2 wt% to 6 wt%, the removal efficiency of IBP had also increased significantly; however, the efficiency dropped as the concentration increased from 6 wt% to 8 wt%. This can be explained based on the transport mechanism in Section 2.4, in which the increase in TOA in the membrane phase caused more IBPH^+^NR_3_ complex to form at the feed-membrane interface. 

The complex was then transported to the membrane-internal interface and reacted with OH^−^ before the IBP was stripped in the internal phase. Thus, this study concluded that the higher the concentration of carrier presented into the membrane phase, the more IBP will be removed. However, too much TOA in the membrane phase caused rapid transfer of the solute from external phase into internal phase which then led to the membrane breakdown [33]. A similar explanation by Chiha et al. reported that more solute was transported from the feed phase, resulting in feed phase dilution and a significant change in the osmotic gradient, causing globules to expand and swell [34]. Thus, 6 wt% was an optimum weight for TOA to obtain higher removal of the IBP from feed phase.

### 3.6. Effect of Surfactant Concentration

**Figure 9:**
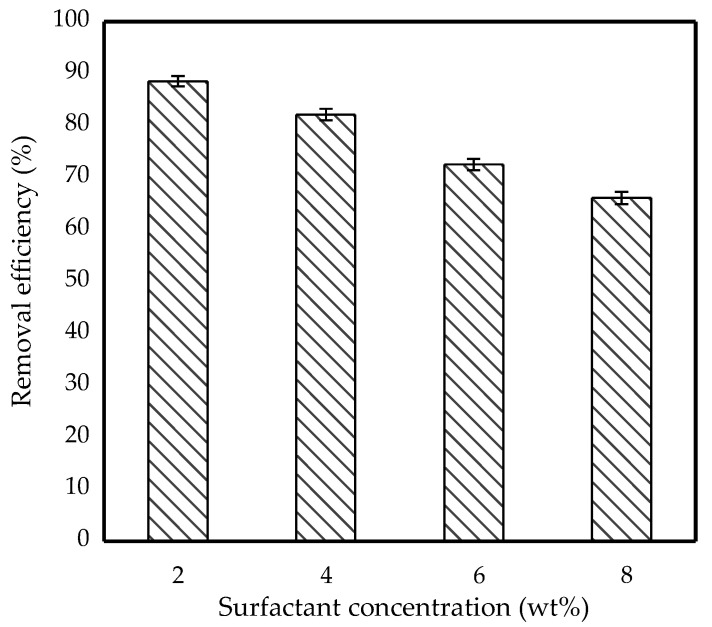
Effect of surfactant concentration on removal efficiency of 10 ppm IBP. (Condition: [TOA] = 6 wt%; Stirring speed = 300 rpm; Emulsification time = 20 min; O/I = 3:1; Kerosene = 30 mL).

The effect of surfactant concentration on IBP removal was varied from 2 wt% to 8 wt%. Figure 9 shows the removal efficiency of IBP against different surfactant concentration. It shows that 89% of the IBP removal efficiency was achieved at a surfactant concentration of 2 wt%.

Uddin et al. found that the surfactant had been adsorbed at the oil–water interface, hence lowering interfacial tension which led to emulsion stabilisation and promoted IBP diffusion through it [35]. However, increasing the Span 80 concentration from 2 wt% to 8 wt% resulted in a reduction in the IBP removal efficiency. This pattern suggested that the surfactant reduced the amount of pollutant transported in the membrane phase because using too much surfactant increased the emulsion viscosity between the phases, hence reducing IBP diffusion [29]. The formation of a more hydrophilic tail on the external-membrane phase increased the surfactant’s hydration capacity, causing the emulsion to swell. Kakoi et al. proposed a similar explanation, claiming that Span 80 has a high proclivity for increasing osmotic swelling [36]. Therefore, 2 wt% of Span 80 was an optimum surfactant concentration in order to give high removal efficiency.

## 4. Conclusions

This study investigated the different parameters affecting the efficiency of 10 ppm IBP extraction. To ensure high ELM performance, all parameters such as TOA (carrier), Span 80 (surfactant), stripping concentration, O/I volume ratio, emulsification time and stirring speed are critical. It was discovered that adding 6 wt% TOA to the membrane caused more IBP to diffuse through the membrane while keeping the Span 80 at 2 wt%. Meanwhile, a 20-min emulsification time with a 300-rpm stirring speed was required to remove at least 89% of the IBP. Furthermore, in comparison to 2:1 and 4:1 O/I ratio, the 3:1 ratio produced the optimum emulsion stability. Kerosene and 0.1 M of NH_3_ were the most promising combination in the ELM’s formulation which resulted in high extraction of IBP. Thus, ELM is proven to be a potential technology for removing low IBP from wastewater owing to its high removal efficiency and product recovery.

## Figures and Tables

**Table 1:** Experimental conditions for the extraction study.

Parameters to Study	Range		Units
	Low	High	
Stirring speed	300	400	rpm
Emulsification time	5	20	min
O/I ratio	2:1	4:1	-
Stripping agent concentration	0.10	0.20	M
Carrier concentration	2	8	wt%
Surfactant concentration	2	8	wt%

## Data Availability

No new data were created or analyzed in this study. Data sharing is not applicable to this article.
